# Pathology and Practice of Medicine

**Published:** 1860-05

**Authors:** Richard J. Dunglison


### PATHOLOGY AND PRACTICE OF MEDICINE.

BY RICHARD J. DUNGLISON, M.D.

I.—*Congenital Inequality of the Two Sides of the Body; with remarkable Physiological Consequences.* By DR. PAUL BROCA, Professeur Agrégé to the Faculty of Medicine, etc., Paris.

Pathological cases sometimes occur which possess so many interesting points, whether studied physiologically or pathologically, that it is difficult to decide to which department their consideration really belongs. But as every marked deviation from the natural condition of a part is a step toward pathology, if not the very first element of it, the singular malformation described by Dr. Paul Broca must be considered under that head, rather than under the subject of physiology. A boy, aged eleven, was presented to his notice, in whom the two halves of the body were unequally developed. The inequality was noticed in infancy, and has appeared to his parents to grow with the child's growth. It is considered, however, as more probable, that the relative difference may have always been the same, although the absolute difference would depend upon the general development. The body of the child is made up of such unsymmetrical halves, that it seems to be the union of parts of two individuals of different ages. The differences in the volumes of the limbs correspond to the differences in length, and are even more decided.

The right lower limb is shorter than the left by five and a half centimètres, (two and one-sixth inches,) the distance being measured from the spine of the ilium to the internal malleolus. From the spine of the ilium to the upper edge of the patella, the difference is nearly an inch greater on the left side. The spine of the ilium of the left side is depressed by nearly two centimètres, (more than three-fourths of an inch,) the depression being unaccompanied by any projection forward or backward, or with any of the deviations consequent on the lengthening or shortening of limbs affected with coxalgia of long standing. It is dependent upon the inequality of development of the two sides. In further illustration of this arrest of development, the right trochanter major is less prominent than the left; and the right half of the scrotum is less voluminous, although no difference is apparent in the size of the testicles. The two halves of the chest are unequal, but no appearance of torticollis is observed.

More curious peculiarities exist in the face, the left eye being more open than the right, and the external commissures of the eyelids being nearer the middle line of the nose on the right than on the left side. The difference is almost three millimètres, (nearly one-eighth of an inch.) Besides other special points of difference in the face, the right half of the cranium is remarkably less developed than the left. A cord placed around the head on a level with the eyebrows, and passing on each side above the ear to meet the external occipital protuberance, measures forty-eight centimètres, (nearly nineteen inches,) twenty-five centimètres (nearly ten inches) belonging to the left side, and twenty-three (nine inches) to the right. The two halves of the skull are, doubtless, unequally developed. Dr. Broca is disposed to think this inequality is not wholly external, and adduces some physiological facts to favor the supposition that it coincides with an analogous arrangement of the two halves of the encephalon. The general sensibility appears to be less fully developed in the right than in the left limbs. The examination of the functions of the organs of sense exhibits that hearing is much more perfect on the left than on the right side; and that the left half of the tongue is very decidedly larger and particularly thicker than the right, the median line being inclined to the right, and the inequality of the two halves being so well marked that an abrupt antero-posterior relief indicates the limits of the left side. This condition resembles closely that which often supervenes as a result of hemiplegia.

The child had already discovered that vision in the right eye was much better than in the left, a condition which was proved by experiments not to be dependent upon myopia of the left eye. The images of the left eye certainly appear to the child to be less distinct and less illuminated than those of the right. Curiously enough, the better eye corresponds exactly to the worse ear, and to the less developed side of the body. Dr. Broca is disposed to consider this inequality of vision as resident neither in the eyeballs nor in the optic nerves, but more posteriorly, either in the optic bands or in their central origin. Much remains, however, to be examined in this case, when the intelligence of the child of maturer years can assist the investigations of the physiologist. The malformation is not hereditary; the family to which he belongs is one remarkably gifted in intelligence, and, although he is perhaps less so than his brothers and sisters were at the same age, yet he is at least as far advanced in this respect as his companions. To relieve the child's lameness, which seems to increase with his growth, gymnastics have been recommended for the especial exercise of the muscles of the right side, with the hope of developing the muscular system rather than of lengthening the limb.

M. Brown-Séquard remarks, in a foot-note, in regard to the employment of galvanism: "The influence of galvanism may even go so far as to induce lengthening of the limbs. I have, in fact, seen in young animals limbs completely paralyzed become, under the action of galvanism, as much developed, with respect to their structure, their dimensions, and the vital properties of their muscles, as healthy limbs."—(*Journal de la Physiologie de l'Homme et des Animaux*, No. 5, January, 1859.)

II.—*Coagulation of the Blood in the Venous System during Life.* By GEORGE MURRAY HUMPHRY, M.D., F.R.S., Lecturer on Anatomy in Cambridge University Medical School.

Several cases of interest having occurred in the practice of Dr. Humphry, of Cambridge, England, he takes the opportunity of describing them in an essay, embracing, as its prominent points, Clots in the Veins, Clots in the Pulmonary Artery, Clots in the Cerebral Sinuses, and Clotting of the Blood in the Cavities of the Heart.

The results of his observations on the first point, *Clots in the Veins*, are, that in almost every case the patients have been previously reduced by some other disease, especially of a chronic form, as phthisis, or discharging abscess, or by old age. In no case had the affection been productive of the alarming symptoms which often attend upon traumatic inflammation of the veins, and which are supposed to depend upon the admixture of purulent or other morbid fluids with the circulating blood. That the presence of clots in the blood depends upon a preternatural tendency to coagulation in the fibrin, Dr. Humphry thinks is confirmed by the fact that obstruction of the vessels commences most frequently in such parts of the venous system as are most favorable to coagulation, such as the great veins of the lower extremity, where the current is more feeble, and especially at the junction of large veins, where the projecting angles furnish favorable spots for the settling of the blood, or in the vicinity of valves, which present edges to which the fibrin may readily adhere. The valves being more numerous in the lower extremities and in the deep veins than in the superficial, would explain why coagulation is of more frequent occurrence in those situations. The uneven surface of the popliteal vein also affords a favorable spot for the formation of clots. Fibrinous deposit and blood-clotting do not go on regularly; hence we have a mottled appearance of the coagula.

The interior of the clot is formed by successive deposits of fibrin; the exterior is often smooth and polished, and adherent to the sides of the vessel. When the clot distends and irritates the walls of the veins, the result is exhibited externally in inflammation of the investing cellular tissue, causing an effusion of serum, lymph, or pus, products which are rarely or never found in the interior. The inner coats of veins do not, indeed, Dr. Humphry thinks, indicate a liability to inflammation, and he is disposed to regard the unfavorable symptoms which occasionally follow ligature of a vein as dependent more upon suppuration of the surrounding cellular tissue. The changes observed in the clot are, sometimes, softening and intimate connection with the walls of the vessel, leading to the complete and permanent obliteration of their canals, or, more commonly, the clots are removed or shrink into delicate bands or fibres, which offer little or no obstruction to the circulation. The affection rarely leads to any serious result; it may be associated with so-called pyæmia, but has no necessary or frequent connection with it.

*Clots in the pulmonary artery* originate from the same general causes and conditions as have been already detailed in the consideration of the subject of clots in the great veins. The clot has not been shown, in a single instance, to be preceded by, or to be attributable to, disease in the coats of the vessel. In some of the illustrative cases here recorded, inflammation, or pulmonary apoplexy, or some other cause, had produced obstruction to the circulation in the pulmonary artery, and thus promoted the settling of the fibrin. Especially is this condition facilitated by the varying condition of the respiration or circulation of the individual, and by the tendency of the blood to coagulate as it approaches the lungs. Dr. Humphry does not think the mode of explanation suggested by Virchow, "that 'emboli' or fragments of clots have been formed in the veins, and been wafted with the blood through the right cavities of the heart, through the lungs," probable in most of the cases. The surface of the venous clots is usually quite smooth, and not so likely, therefore, to be disintegrated by the slowly-flowing current of blood; and the effects of a preternatural tendency of the venous blood to coagulate are just as likely to be exhibited in the pulmonary arteries as elsewhere.

*Clots in the cerebral sinuses* are described in the case of a young girl, who died after a curious nervous attack, attended with inability to move the left arm and leg, and afterwards with drowsiness and stupor. Besides other appearances revealed on post-mortem examination, the venæ Galeni were found greatly distended with firm, mottled clots, and the same condition existed in the right cavernous, the straight, petrosal, and lateral sinuses. Some of the veins on the surface of the hemisphere were blocked up with mottled coagula. The blocking up of the sinuses was probably the earliest of the morbid phenomena, the others being dependent upon it. It does not seem so clear, however, why a deposition of fibrin should have taken place in these vessels.

*Clots in the cavities of the heart* form the most interesting feature, as well as the most important pathological element, in a case whose post-mortem appearances are carefully described. Death from clots filling the cavities of the heart, and all the vessels connected with the right side and the left ventricle, was clearly indicated. Many of the phenomena detected in such cases are difficult to explain, whether as changes taking place after or before death. Fibrinous clots frequently fill up the cavities of the heart, and are prolonged into the adjacent vessels, and extend into the most dependent branches of the pulmonary arteries, the lowermost portions of the clots being as devoid of color as the uppermost. Yet it seems pretty certain that clots of this kind are, for the most part, formed after death. Dr. Humphry differs from Dr. Richardson, in his remarks "On the Coagulation of the Blood," in the view that the tubular or the laminated structure of a clot, or that a clot consisting of a fibrinous tube containing red blood in its interior, is conclusive evidence of the coagulation having begun before death. Under favorable circumstances, the lamination may be effected by peculiar disposition of the fibrin in plates or layers after death; and the same fact may be asserted in regard to the tubular form of the clot. The same post-mortem tendencies may apply also to the deposition of fibrinous clots on and near the valves, on the interior of the vessels, and between the columnæ carneæ of the heart.

In this way a tubular lining of fibrin may be laid down in the interior of the pulmonary artery after death, and be moulded upon the sigmoid valves, while the remainder of the blood in the vessels is fluid. This remaining blood subsequently coagulating would form a soft, dark, cylindrical clot, inclosed in and continuous with the fibrinous tube. Still later the contraction of the whole clot, more particularly of the outer fibrinous tube, would draw it away from the sides of the vessel and leave it free in the interior, or nearly so. Thus, by a sequel of intelligible *post-mortem* processes we may have produced those tubular fibrinous clots containing red, clotted blood in their interior, as well as those fibrinous or parti-colored strings between the columnæ carneæ, which have been described by Dr. Richardson and others, and which are commonly thought to be dependent upon changes going on during life. Such clots are not always formed after death, but they may be so; and care must be taken, therefore, how we admit these and other similar appearances to be evidence that the coagulation of the fibrin has taken place during life.

We have purposely omitted the details of a number of interesting cases under each prominent point which we have considered, and have preferred to give a sketch of Dr. Humphry's views. The subject is certainly a suggestive one, on which much light may yet be thrown by careful observers, among whom the author of the essay we have been considering may well be placed.—(*From the British Medical Journal, and a reprint in pamphlet form*, pp. 42; Cambridge, 1859.)

III.—*A Case Illustrating the Pathology of the Cerebellum.* By G. ROLLESTON, M.D., F.R.C.P., etc.

Two symptoms of cerebellar disease have been observed in several recently recorded cases, which, when combined, are considered by Dr. Rolleston as pathognomonic. The first is an inability to maintain the erect posture when raised into it, coupled with complete power over either or both of the legs when in the horizontal posture; the second, stiffness of the neck. An illustrative case is furnished by him, and he attempts to show that "what we know alike of the pathology and of the physiology of the cerebellum is clearly explicable on the hypothesis that that great nerve-centre stands in the same relation to the motor nerve nuclei of the trunk and (posterior?) limb muscles, as the corpora olivaria do to the motor nerve nuclei of the face, tongue, and throat muscles." The corpora olivaria, it may be remarked, have been considered by some observers as agents modifying the impression which they have received from the medulla oblongata, so as to call forth, when reflected on to motor nuclei, and down motor fibres, the required bilateral muscular action. The patient, a child ten years of age, was seized, early in 1859, with a "bad cold in the head," and with chills. A week or two afterwards she could not maintain the erect position, although she had the power of moving her legs when lying in bed. She suffered from rheumatic fever, and in about three months or more after the first attack had two convulsive fits, after which she began to recover the use of her lower limbs, and to sit up. She could always use her hands and arms.

As described by Dr. Rolleston, nearly nine months after the appearance of her first symptoms, she can turn her head from side to side on the pillow, not, however, without considerable difficulty in accomplishing it, while she catches at her head and supports it in her hands, if she be raised up in bed. If the head be left unsupported, it falls to one side or the other. She can walk along by the side of a wall or table, provided she can get some support for her head, and the hand and arm she rests it upon. She has stiffness of the neck, but not of both sides of the neck at the same time. When the head falls over, it does so with much greater force than its mere weight would account for. Dr. Rolleston considers that "for the support of the head, consentaneous and bilateral muscular action is required; but when the functions of the cerebellum are in abeyance, this result is not obtainable even in obedience to the orders of the will. But no bilateral action is necessary for the turning of the head from side to side, if the back of the head lies supported on the pillow. This movement she can execute, therefore, just as formerly she could move either leg while lying horizontally, though unable to maintain the erect posture." It is conjectured that the meninges of the cerebellum were first affected, although the unavoidably imperfect history of the case interferes with the expression of a certainty in the pathology. —(*London Medical Times and Gazette*, Feb. 18, 1860.)

IV.—*Phthisis of the French Millstone-makers.* By Thomas B. Peacock, M.D., F.R.C.P., etc.

Of the influence of certain occupations as exciting causes of disease, and especially of the prevalence of tubercular consumption among those engaged in some trades more than in others, much has been written and observed of late years. As long ago as 1727, Wepfer noticed the frequent occurrence of phthisis among millstone-cutters on the Rhine; while in 1799, Dr. Johnstone described to the Medical Society of London numerous cases of "grinder's rot," as consumption among the needle-pointers of Worcestershire was locally called. Many other observers have noticed similar facts, and the experience of medical men among the workers in stone and metal in Edinburgh and Sheffield long since established the fact, that those occupations in which the particles of stone or metal are thrown off, and inhaled by the workpeople, exert a very injurious influence on their health. Dr. Peacock draws attention to the prevalence of pulmonary disease in a class of workmen among whom, he thinks, it has not been hitherto particularly noticed—the French millstone-makers or builders.

These men work upon a peculiarly hard kind of flint, known in the trade as the "French burr." Every stroke of the chisel is attended by a bright flash of light and a cloud of dust, and larger or smaller particles of stone, forming a sharp grit, are thrown off. Portions of the stone and of iron from the chisel not unfrequently become imbedded in the hands or faces of the workmen, so that the backs of the hands of those who have been long at the trade are studded with small bluish spots. Dr. Peacock was informed by the foreman of one of the yards, that he had known at least twenty persons die of chest affections out of the small number, not, perhaps, exceeding fifty, who were employed at the work in London, the oldest workmen not having been employed more than thirteen years. He notes the difference between men thus engaged, and those who in the same establishment are occupied in wire-weaving, etc. The results arrived at by him may be thus arranged:—

The average age of wire-weavers was 35·84 years; of millstone-cutters, 24·1 years. The ages of the five oldest wire-weavers were 71, 43, 42, 40 and 40; while the five oldest among the millstone-cutters were still very young men, their ages being 38, 29, 29, 28 and 28. The average period during which the former class had worked at the trade was 20·69 years, and eight of them had been engaged for 51, 32, 29, 25, 25, 24, 24 and 22 years; while the average period during which the millstone-cutters had worked was but 8·9 years, and the three who had been longest engaged had been 18, 17, and 14¾ years. Yet the wire-weavers, as a class, are stated to be less healthy-looking than the millstone-cutters, although enjoying in the main much better health. Workers in metal, too, must labor under less favorable hygienic circumstances than the other class, for their occupation confines them more steadily indoors.

Besides the irritating effects of the fine dust upon the air-passages, other causes of deterioration of health also exist, such as working in damp underground cellars or open sheds exposed to the weather, the difficulty of expanding the chest while leaning over the millstone, and the irregular habits of the workmen. The men are accustomed to take large quantities of beer and spirits in the course of the day, four or five pints of beer being by no means an unusual allowance. Dr. Peacock does not consider the causes named sufficient to explain the great tendency to pulmonary affection among the millstone-makers, apart from the injurious influence which is exercised by the gritty particles of silex which they inhale while at work. This, he considers, is the main cause of their sufferings.

The history of two cases of phthisis is given, and the post-mortem examinations are carefully detailed. In one of the cases, in which the diseased portions of lung were greatly indurated, little or no trace of original lung-structure was found, upon microscopic examination, to exist. The diseased masses appeared to be made up of dense, closely-arranged fibroid tissue, studded here and there with numerous irregular groups of black pigment, and generally with an abundance of transparent granules and globules of various sizes. When the indurated tissue was exposed to heat and nitric acid, a considerable quantity of gritty matter remained, which was inferred to be siliceous. Dr. Peacock considers it probable that in persons who become phthisical from being exposed to local irritation from the inhalation of particles of stone or metal, the disease will be slower in its progress, and be preceded by more marked symptoms of faucial and laryngeal irritation than under other circumstances.

The work is much less injurious to those who take to it when more advanced in life than to those who enter it in youth. Means are suggested by Dr. Peacock to improve the health of the men, which, if properly attended to, would doubtless prolong the lives of the millstone-builders. The avoidance of damp underground cellars, the upright instead of the stooping position, the working of wet instead of dry stones, and the employment of respirators to protect the air-passages, are the means suggested to mitigate the injurious effects of this occupation.—(*British and Foreign Medico-Chirurgical Review*, Jan., 1860.)

V.—*Small-pox in Scotland, as it is, was, and ought to be.* By ALEXANDER WOOD, M.D., President of the Royal College of Physicians, Edinburgh.

Whenever statistics are carefully collected, they become valuable sources of information, especially in pathology, when it is practicable to compare the results of different modes of preventive or curative treatment. Especially are they interesting when considered in relation to such a scourge as small-pox, whose annual ravages in Europe alone at the close of the last century have been estimated at half a million of lives. To explain and correct an erroneous impression in regard to the spread of insanity, Dr. Wood considers the past and present history of the disease in Scotland, and furnishes useful hints for future protection from it. The inferences he deduces on the present history of small-pox are—

1. That the disease is not steadily increasing year by year, but that it fluctuates in frequency and fatality just as it did before the discovery of vaccination.

2. That at one time it prevails extensively in one town; at another it leaves it and ravages another. In 1856, Dundee, and in 1857, Glasgow and Leith, were the towns principally affected by it.

3. That taking the year 1856, when small-pox was epidemically present, out of a total estimated population of 854,066 individuals, 645 died of small-pox.

4. That the total mortality from all causes being in that year 22,248, the deaths from small-pox (645) thus constituted 2·8 per cent., which is double the average of London for the last ten years, or of England and Wales for the last seven, and fourteenfold the average of Bohemia and Lombardy.

Dr. Wood shows how small-pox, as controlled by vaccination, possesses the same tendency that it did when it presented its severest characters, to rapid and sudden alterations of prevalence. The total deaths from it in Scotland for 1856, for example, were 645, while in 1858 they were only 175, and in 1859, 466. In the returns collected by the Faculty of Medicine of Prague, we find additional confirmation of the fact. In 1797, before vaccination, of 86,885 deaths, 1988 were from small-pox, while, in 1799, 17,587 deaths from that disease occurred in a total mortality of 99,079. After vaccination, 62 deaths from small-pox occurred in 108,419 deaths in the year 1838, while in 1840 the number was 699 in a total of 118,471 deaths.

Of *small-pox as it was*, previous to the common use of vaccination, statistical figures seem to indicate a percentage of deaths, in the total mortality, of about 18. Instead of 645 deaths in 1856, the number should have been, according to this proportion, 4003 deaths. The disease formerly attacked a very different class in the social scale from those who become its victims at the present day. Severe attacks are now unfrequent, and deaths exceedingly uncommon among the upper classes of society; and the disease, when it does occur among them, is usually of that modified form which it assumes when it attacks those who have been vaccinated.

Of *small-pox as it ought to be*, Dr. Wood questions whether the maximum of benefit which vaccination is capable of conferring has yet been attained. Of his remarks on the comparative value of inoculation and vaccination, we need not speak, except to state that the Report of the Epidemiological Society (*Brit. and For. Med.-Chirur. Review*, vol. xx.) exhibits that, during 63 years, in which inoculation was practiced, there was a ratio of 84 epidemics in 100 years, while during the last 50 years (from 1807) the ratio is 24 epidemics in 100 years.

Dr. Wood considers it very doubtful whether small-pox will ever be entirely exterminated by general vaccination, because the disease spreads epidemically as well as by contagion; because there are individuals to whom neither a previous attack nor vaccination affords immunity from a subsequent attack of the disease; because the mortality from small-pox in the unvaccinated, taken generally, is 35 per cent. of those attacked; but of children under five years of age, it is 50 per cent., the mortality among the vaccinated attacked by small-pox being 7 per cent. taken generally; among the badly vaccinated, it is 15 per cent.; among the well vaccinated, 1 per cent.

The deaths from small-pox in Scotland are never less than 2 per cent. of the whole annual mortality in the eight large towns, and in January, 1856, were as great as 30 per cent. in one of them. The suggestions of Dr. Wood in regard to legislative interference in the matter of vaccination, gave rise to considerable discussion in the Medico-Chirurgical Society of Edinburgh, but the plans recommended are more of local than of general interest.—(*Edinburgh Medical Journal*, February, 1860.)

VI.—*Œsophageal Fistula communicating with the Exterior of the Right Side of the Chest, through the Lungs and Pleural Cavity.* By CHARLES F. MACLACHLAN, M.D.

Dr. Maclachlan furnishes the details of a case, which he very properly regards more as a medical curiosity than as having much practical value, except as an additional evidence that a great amount of disease may exist and be compatible with life and sometimes with comparative ease. The patient, a young woman, twenty-five years of age, became subject, about eleven years ago, to severe pain in the right infraclavicular region, shooting down the arm and forearm. In 1857 she had an attack of hæmoptysis, with pleuritic pain at the lower part of the region just mentioned. In the spring of 1858 she complained of considerable pain in the neighborhood of the right nipple, and in May an abscess was discovered, pointing externally, slightly below and to the left of the same nipple. Slight emphysema existed at some parts of the surface, and the pain was intense. A large quantity of offensive pus and some air were discharged from the abscess when it was opened, and great relief followed for awhile. Pain in the throat soon supervened, however, just below the larynx, at the "swallow," as she expressed it; and, in a few weeks afterwards, she noticed particles of oatmeal and of food in the matter discharged from the abscess and on the dressings. Long pieces of lymph were discharged from the abscess for several months afterwards, their passage being accompanied with a great increase of the local pain.

Her present condition, as described by Dr. Maclachlan, is interesting. Very little food passes through the fistulous opening when she remains in a recumbent position. Warm gruel and milk appear externally at the orifice immediately after being swallowed, if she be seated in an upright posture. She has suffered from rheumatism and dysentery while confined to bed; her breathing has been comfortable ever since the abscess was opened; she has no cough or expectoration; the same kind of pain in the right breast annoys her, and her feet and legs are œdematous. The local appearances are, three fistulous openings in a row, one above the other, commencing about two inches above and to the left of the nipple; the lowest one, from which the discharge comes, being about one inch below the level of the nipple. Percussion indicates unusual clearness of the right side of the chest, especially at the upper part, anteriorly and posteriorly; and in these situations, metallic tinkling, amphoric resonance, and gurgling are heard, indicative of a large cavity containing fluid and air.

Dr. Maclachlan suggests that an abscess of the lung, opening into the pleural cavity, was the primary cause of the abnormal condition now existing, and expresses the belief that the opening into the œsophagus must be beyond the bifurcation of the bronchi, as no appearances have existed to indicate a fistulous tract running through the soft parts of the neck in the neighborhood of the trachea, toward the pleural cavity.

Dr. Maclachlan does not give any prognosis in regard to the future history of the case; we trust, however, that he may hereafter record any changes of interest, which he may find in its progress toward recovery, or a reverse condition.—(*Glasgow Medical Journal*, January, 1860.)

VII.—*Virchow's Views on Cellular Pathology.* By G. H. E. Baumgarten, M.D.

The researches of Professor Rudolphus Virchow on "Cellular Pathology, based on Physiological and Pathological Histology," have not been accessible to the great majority of the profession, on account of the German garb in which they were clothed. In the *British and Foreign Medico-Chirurgical Review* for October, 1859, his doctrines were criticised and dwelt upon analytically by one who appreciated their weakest points as well as their strongest claims to credence. We condense what Dr. Baumgarten has furnished to American readers as his view of Virchow's pathological theories. The basis of the theory of cellular pathology is the principle that a single cell or group of cells, operated upon by the same cause of disease, may be altered chemically, morphologically, and functionally, without any primary affection of the blood or the nervous system. As every individual cell is supposed to have an individual function, it is inferred that the function may become deranged, and disease result, and that each cell may become diseased alone, without pre-existing disease in its vicinity. With this groundwork for a general doctrine of cellular pathology, the views of Virchow are extended to embrace a wide field of scientific theorizing, much of which is doubtless true, but much must also be hypothetical.

After defining the distinctions between an animal cell and a vegetable cell, the importance of the intercellular substance is alluded to, and from the different relations which the cells bear to each other and to the intercellular substance, the mode in which tissues are built up is explained. The group of tissues in which cells have taken a specific development comprises those which are essential for the character of an animal, as nerve, muscle, and the vascular system. Those tissues in which the cell not only labors for its own nutriment, but also furnishes a certain quantity of substance outside of its walls, comprise the connective tissue and all that is related to, or developed from it, including areolar tissue, cartilage, mucous tissue, adipose tissue, and bone. Pathological formations may be classified on the same principles as the normal tissues just referred to, and, in this sense, heterology of pathological products does not exist. *Heterological* formations, they can be called, only so far as they have elements analogous to some physiological formation, existing on a spot where they ought not to grow, (*heterotopical,*) or at a time when they should not exist, (*heterochronical,*) or only in a greater quantity than is proper, (*heterometrical developments.*) The physiology of the nutritive process must be understood before the pathological condition can be thoroughly intelligible.

In the processes of active hyperæmia, the muscular elements of the artery are essentially active, relaxation speedily following irritation. The dilatation of the vessel depends in all cases on a sort of paralysis of the muscular coat, and "active hyperæmia," so called, consequently does not depend on any vascular action. The greater or less quantity of blood which flows through a part is not the cause of difference in nutrition. If we diminish the quantity of nutritive material, we can prevent a part from absorbing much, but it does not follow from this, that we can compel a part to assimilate more material by offering more blood to it. Nor does an increase in the nutritive processes of a part depend so much on a greater amount of blood in general or in the part itself, as on certain states of the tissues, (*irritation,*) which change its affinities to the constituents of the blood, or on the presence of specific substances in the latter, which are particularly attracted by certain parts of the tissues. An element, destined to attract substances from the blood, must be intact; any alteration of its molecular, physical, or chemical properties, by disease, will alter the power of executing this attraction. Cellular pathology merely demands that this law, so true in regard to the larger organs, should be admitted as equally applicable to the smaller organs and elements; that it be conceded that an epidermic cell, a cartilage cell, etc., also possesses in some measure the power of attracting materials from the blood according to its wants. The various distinctions between the humoro-pathological doctrine and those of the cellular pathology are pointed out, especially in the view of the blood being an independent formation, regenerating itself by its own resources. The theory of Virchow denies this, and considers the blood as constantly dependent on other organs. The history of the pathology of pyæmia, embolia, and the chemical dyscrasiæ, properly form a part of this branch of the subject, which is discussed at length in the original paper of Virchow. In his theory of independent cell-action, and in his refutation of the doctrine of the neuro-pathologist, he remarks that "every element can be excited to action without any influence or mediation of the nervous system;" and this observation is extended to the different action of different irritants on nearly all excitable parts.

In the limits of this abstract, we can, in addition to the points already considered, merely refer to the remaining subjects of Dr. Baumgarten's analysis of Virchow's views. These are, the phenomena of nutritive irritation, increased assimilation from a direct external irritant, formation, changes in the development of cells, the definition and pathology of inflammation in all its manifold phases, the difference between normal and pathological developments, the formation of mucus and pus, and a number of other materials for reflection and sober consideration. To these are added Dr. Baumgarten's recollections of other views of Virchow, as contained in his lectures and writings. The subject is so minutely histological in its details, that a good deal of the interest which might otherwise be felt in it is lost to those who may find a difficulty in comprehending the refinements of division in which the theory indulges.—(*St. Louis Medical and Surgical Journal*, January, 1860.)

